# Right Parotid Fibrolipoma: A Rare Lesion in a Child

**DOI:** 10.21699/ajcr.v7i4.448

**Published:** 2016-09-01

**Authors:** Kamal Nain Rattan, Sunita Singh, Shruti Bansal

**Affiliations:** 1 Department of Pediatric Surgery, Pt. B.D. Sharma PGIMS Rohtak, Haryana; 2 Department of Pathology, Pt. B.D. Sharma PGIMS Rohtak, Haryana

**Keywords:** Lipoma, Parotid gland, Child

## Abstract

Lipoma rarely involves parotid gland especially in children. An 11-year-old boy presented with right parotid swelling. Preoperative workup including CT scan and FNAC gave suspicion of parotid gland lipoma. The diagnosis was confirmed on histopathology after complete excision of the mass.

## CASE REPORT

An 11-year-old boy presented with a slowly progressive painless swelling in the right parotid region for the last 10 years (Fig.1). Physical examination revealed firm well defined non tender swelling measuring 3.5 cm × 3.5cm in the right parotid region. The overlying skin was normal and there was no cervical lymphadenopathy. Facial nerve function was also intact. A high frequency ultrasonography visualized hyperechoic lesion (2.8cm × 1.1cm) in the subcutaneous plane. CT scan showed a fat density lesion measuring approximately 3.3cm × 2.4cm × 4cm, in right parotid gland. Fine needle aspiration cytology (FNAC) was conclusive for the presence of mature fibroadipose tissue fragments consistent with lipoma. At surgery, the mass was encapsulated, soft, yellow and fatty, located in the superficial lobe of right parotid gland (Fig.1). Superficial parotidectomy with lipoma excision was done without injury of any major neurovascular structures (Fig.2). The drain was kept insitu for 48 hours. The specimen was sent for histopathological examination and the diagnosis was confirmed as fibrolipoma, a histological variant of lipoma (Fig.2). Postoperatively, neuropraxia of facial nerve was noticed from which the patient is improving and is doing well in follow up.

**Figure:**
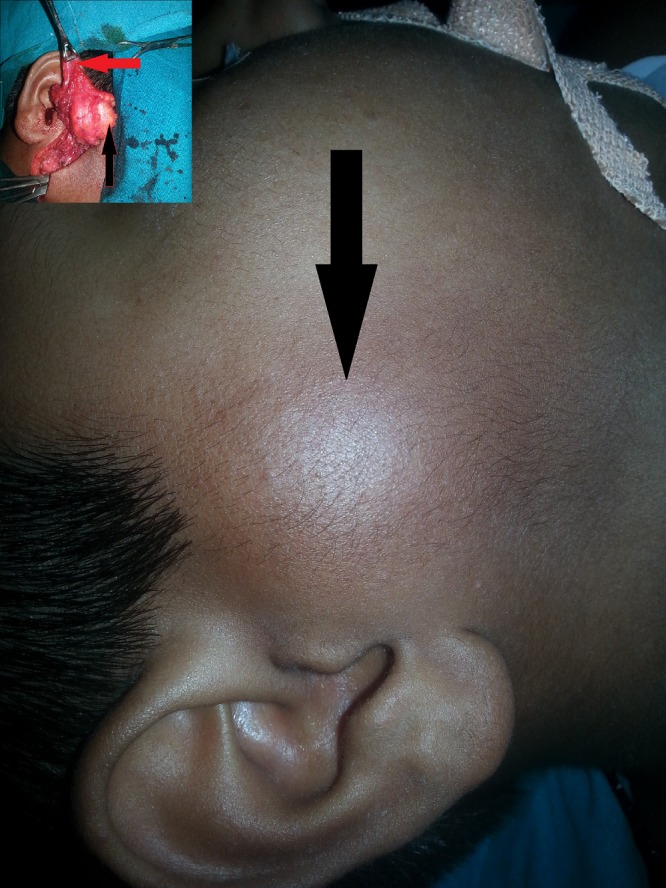
Figure 1: Photograph of a 11 year old male child who presented with 10 year history of slow growing swelling in right parotid region. Inset shows intraoperative picture with raised skin flap(red arrow) and lipoma in superficial lobe of right parotid (black arrow).

**Figure:**
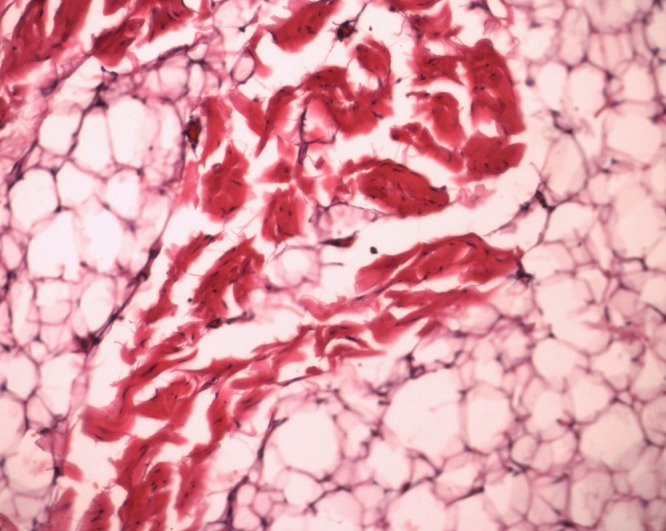
Figure 2:Histopathology of fibrolipoma (H and E stain, ×100).

## DISCUSSION

Lipoma of parotid gland is quite rare in children with an incidence of 4.4%.[1] Parotid lipomas usually develop in the fifth and sixth decades with a definite male predominance.[2] Only few case reports of pediatric parotid gland lipoma have been published in literature.[2,3] The etiology of pediatric parotid lipoma remains unclear. In 75% of cases, parotid lipomas occur in the superficial lobe while the deep lobe is involved in only 8.5% of cases and both the lobes are involved in 16.5% of cases.[4] Lipomas can be single or multiple. But the majority of children usually have a single lesion as found in our case. Lipomas exhibit morphological variants which include fibrolipoma, myxolipoma, etc.[2,3,5] In the index case it was fibrolipoma on histopathological examination.

Clinically, the parotid lipoma appears as soft, slow growing, painless and well defined mass.[2,5] In our case too, it was noted for 10 years by parents before the child presented to us. Preoperative imaging, like CT scan and MRI, plays an important role in diagnosis.[1] FNAC can also give clue however, excision biopsy with histopathological examination remains gold standard for ultimate diagnosis. Histologically, lipomas exhibit resemblance to normal mature adipose tissue. They are distinguished from normal simple fat aggregation by the presence of a fibrous capsule.[6] Surgical excision with facial nerve preservation remains the mainstay of treatment but is quite challenging and should be done by experienced surgeons. In our case despite meticulous dissection and preservation of facial nerve, neuropraxia of facial nerve was observed postoperatively which improved on conservative measures.

In conclusion, being a rare neoplasm, parotid lipoma is seldom considered in the initial differential diagnosis of parotid mass. Its possibility should be kept in mind when dealing with slow growing painless parotid masses in pediatric age group.

## Footnotes

**Source of Support:** Nil

**Conflict of Interest:** None declared

